# Differential equation and inequalities of the generalized k-Bessel functions

**DOI:** 10.1186/s13660-018-1772-1

**Published:** 2018-07-16

**Authors:** Saiful R. Mondal, Mohamed S. Akel

**Affiliations:** 1 Department of Mathematics and Statistics, College of Science, King Faisal University, Al-Hasa, Saudi Arabia; 2 Department of Mathematics, Faculty of Science, South Valley University, Qena, Egypt

**Keywords:** 33B15, 33C10, 26A48, 26D07, k-Gamma, k-Bessel, Turán-type inequality, Monotonicity

## Abstract

In this paper, we introduce and study a generalization of the k-Bessel function of order *ν* given by

$$ \mathtt{W}^{\mathtt{k}}_{\nu , c}(x):= \sum_{r=0}^{\infty } \frac{(-c)^{r}}{\Gamma_{\mathtt{k}} ( r \mathtt{k} +\nu +\mathtt{k} ) r!} \biggl( \frac{x}{2} \biggr) ^{2r+\frac{\nu }{\mathtt{k}}}. $$

We also indicate some representation formulae for the function introduced. Further, we show that the function $\mathtt{W}^{ \mathtt{k}}_{\nu , c}$ is a solution of a second-order differential equation. We investigate monotonicity and log-convexity properties of the generalized k-Bessel function $\mathtt{W}^{\mathtt{k}} _{\nu , c}$, particularly, in the case $c=-1$. We establish several inequalities, including a Turán-type inequality. We propose an open problem regarding the pattern of the zeroes of $\mathtt{W}^{ \mathtt{k}}_{\nu , c}$.

## Introductions

Motivated with the repeated appearance of the expression

$$ x(x + \mathtt{k}) (x + 2\mathtt{k})\cdots \bigl(x + (n -1)\mathtt{k} \bigr) $$

in the combinatorics of creation and annihilation operators [13, 14] and the perturbative computation of Feynman integrals (see [12]), a generalization of the well-known Pochhammer symbols is given in [15] as

$$ (x)_{n,\mathtt{k}}:=x(x + \mathtt{k}) (x + 2\mathtt{k})\cdots \bigl(x + (n -1) \mathtt{k} \bigr), $$

for all $\mathtt{k}>0$, calling it the Pochhammer k-symbol. Closely associated functions that have relation with the Pochhammer symbols are the gamma and beta functions. Hence it is useful to recall some facts about the k-gamma and k-beta functions. The k-gamma function, denoted as $\Gamma_{\mathtt{k}}$, is studied in [15] and defined by

1.1 $$\begin{aligned} \Gamma_{\mathtt{k}}(x):= \int_{0}^{\infty }t^{x-1}e^{-\frac{t^{ \mathtt{k}}}{\mathtt{k}}}\,dt \end{aligned}$$

for $\operatorname {Re}(x)>0$. Several properties of the k-gamma functions and applications in generalizing other related functions like k-beta and k-digamma functions can be found in [15, 27, 28] and references therein.

The *k*-digamma functions defined by $\Psi_{\mathtt{k}}:= \Gamma_{\mathtt{k}}'/\Gamma_{\mathtt{k}} $ are studied in [28]. These functions have the series representation

1.2 $$\begin{aligned} \Psi_{\mathtt{k}}(t):=\frac{\log (\mathtt{k})-\gamma_{1}}{\mathtt{k}}- \frac{1}{t} +\sum _{n=1}^{\infty }\frac{t}{n\mathtt{k}(n\mathtt{k}+t)}, \end{aligned}$$

where $\gamma_{1}$ is the Euler–Mascheroni constant.

A calculation yields

1.3 $$\begin{aligned} \Psi_{\mathtt{k}}'(t)=\sum _{n=0}^{\infty } \frac{1}{(n\mathtt{k}+t)^{2}}, \quad \mathtt{k}>0 \mbox{ and } t>0. \end{aligned}$$

Clearly, $\Psi_{\mathtt{k}}$ is increasing on $(0, \infty )$.

The Bessel function of order *p* given by

1.4 $$ \mathtt{J}_{p}(x):= \sum_{k=0}^{\infty } \frac{(-1)^{k}}{\Gamma { ( k +p+1 ) } \Gamma { ( k+1 ) }} \biggl( \frac{x}{2} \biggr) ^{2k+p} $$

is a particular solution of the Bessel differential equation

1.5 $$\begin{aligned} x^{2} y''(x)+ x y'(x)+ \bigl(x^{2}-p^{2} \bigr)y(x)= 0. \end{aligned}$$

Here Γ denotes the gamma function. A solution of the modified Bessel equation

1.6 $$\begin{aligned} x^{2} y''(x)+ x y'(x)- \bigl(x^{2}+{\nu }^{2} \bigr)y(x)= 0, \end{aligned}$$

is the modified Bessel function

1.7 $$ \mathtt{I}_{\nu }(x):= \sum_{k=0}^{\infty } \frac{1}{\Gamma { ( k +\nu +1 ) } \Gamma { ( k+1 ) }} \biggl( \frac{x}{2} \biggr) ^{2k+\nu }. $$

The Bessel function has several generalizations (see, e.g., [9, 10]) and is notably investigated in [1, 17]. In [1], a generalized Bessel function is defined in the complex plane, and sufficient conditions for it to be univalent, starlike, close-to-convex, or convex are obtained. This generalization is given by the power series

1.8 $$\begin{aligned} \mathcal{W}_{p, b, c}(z)= \sum_{k=0}^{\infty } \frac{(-c)^{k} ( \frac{z}{2} ) ^{2k+p+1}}{\Gamma ( k+1 ) \Gamma ( k+p+ \frac{b+2}{2} ) },\quad p, b, c \in \mathbb{C}. \end{aligned}$$

In this paper, we consider the function defined by the series

1.9 $$\begin{aligned} \mathtt{W}_{\nu , c}^{\mathtt{k}} (x) :=\sum _{r=0}^{\infty }\frac{(-c)^{r} }{ \Gamma_{\mathtt{k}}(r \mathtt{k}+ \nu + \mathtt{k}) r!} \biggl( \frac{x}{2} \biggr) ^{2r+\frac{ \nu }{\mathtt{k}}}, \end{aligned}$$

where $\mathtt{k}>0$, $\nu >-1$, and $c \in \mathbb{R}$. As $\mathtt{k}\to 1$, the k-Bessel function $\mathtt{W}_{ \nu , 1}^{1}$ is reduced to the classical Bessel function $J_{\nu }$, whereas $\mathtt{W}_{\nu , -1}^{1}$ coincides with the modified Bessel function $I_{\nu }$. Thus, we call the function $\mathtt{W}_{\nu , c} ^{\mathtt{k}}$ the generalized k-Bessel function. Basic properties of the k-Bessel and related functions can be found in recent works [8, 19–21].

Turán [30] proved that the Legendre polynomials $P_{n}(x)$ satisfy the determinantal inequality

1.10 $$\begin{aligned} \biggl\vert \textstyle\begin{array}{@{}c@{\quad }c@{}} P_{n}(x) & P_{n+1}(x) \\ P_{n+1}(x) & P_{n+2}(x) \end{array}\displaystyle \biggr\vert \leq 0, \quad -1 \le x \le 1, \end{aligned}$$

where $n = 0, 1, 2, \ldots $ , and the equality occurs only for $x = \pm 1$. The inequalities similar to (1.10) can be found in the literature [2, 3, 5, 11, 16, 25] for several other functions, for example, ultraspherical polynomials, Laguerre and Hermite polynomials, Bessel functions of the first kind, modified Bessel functions, and the polygamma function. Karlin and Szegö [24] named determinants in (1.10) as Turánians. More details about Turánians can be found in [5, 11, 18, 22, 23, 29].

The aim of this paper is to investigate the influence of the $\Gamma_{\mathtt{k}}$ functions on the properties of the k-Bessel function defined in (1.9). It is shown that the properties of the classical Bessel functions can be extended to the k-Bessel functions. Moreover, we investigate the effects of $\Gamma_{\mathtt{k}}$ instead of Γ on the monotonicity and log-convexity properties and related inequalities of the k-Bessel functions. The outcomes of our investigation are presented as follows.

In Section 2, we derive representation formulae and some recurrence relations for $\mathtt{W}_{\nu , c}^{\mathtt{k}}$. More importantly, the function $\mathtt{W}_{\nu , c}^{\mathtt{k}}$ is shown to be a solution of a certain differential equation of second order, which contains (1.5) and (1.6) for the particular case $\mathtt{k}=1$ and for particular values of *c*. At the end of Section 2, we give two types of integral representations for $\mathtt{W}_{\nu , c}^{\mathtt{k}}$.

Section 3 is devoted to the investigation of monotonicity and log-convexity properties of the functions $\mathtt{W}_{\nu , c} ^{\mathtt{k}}$ and to relation between two k-Bessel functions of different order. As a consequence, we deduce Turán-type inequalities.

In Section 4, we give concluding remarks and list two tables for the zeroes of $\mathtt{W}^{\mathtt{k}}_{\nu , c}$, leading to an open problem for future studies.

## Representations for the k-Bessel function

### The k-Bessel differential equation

In this section, we find differential equations corresponding to the functions $\mathtt{W}_{\nu , c}^{\mathtt{k}}$.

#### Proposition 2.1

*Let* $\mathtt{k}>0$ *and* $\nu >-k$. *Then the function* $\mathtt{W}_{ \nu , c}^{\mathtt{k}}$ *is a solution of the homogeneous differential equation*

2.1 $$\begin{aligned} x^{2} \frac{d^{2}y}{dx^{2}}+ x \frac{dy}{dx}+ \frac{1}{\mathtt{k}^{2}} \bigl( c x^{2} \mathtt{k}- {\nu^{2}} {} \bigr) y=0. \end{aligned}$$

#### Proof

Differentiating both sides of (1.9) with respect to *x*, it follows that

$$\begin{aligned} \frac{d}{dx}\mathtt{W}_{\nu , c}^{\mathtt{k}} (x) =\sum _{r=0}^{\infty }\frac{(-c)^{r} ( 2r+\frac{\nu }{\mathtt{k}} ) }{ \Gamma_{\mathtt{k}}(r \mathtt{k}+ \nu + \mathtt{k}) r!} \biggl( \frac{x ^{2r+\frac{\nu }{\mathtt{k}}-1}}{2^{2r+\frac{\nu }{\mathtt{k}}}} \biggr) . \end{aligned}$$

This implies

2.2 $$\begin{aligned} x\frac{d}{dx}\mathtt{W}_{\nu , c}^{\mathtt{k}} (x) = \sum_{r=0}^{ \infty }\frac{(-c)^{r} ( 2r+\frac{\nu }{\mathtt{k}} ) }{ \Gamma_{\mathtt{k}}(r \mathtt{k}+ \nu + \mathtt{k}) r!} \biggl( \frac{x}{2} \biggr) ^{2r+\frac{\nu }{\mathtt{k}}}. \end{aligned}$$

Now differentiating (2.2) with respect to *x* and then using the property $\Gamma_{\mathtt{k}}(z+\mathtt{k})= z \Gamma_{\mathtt{k}}(z)$ of the k-gamma function yield

$$\begin{aligned} &x^{2} \frac{d^{2}}{dx^{2}}\mathtt{W}_{\nu , c}^{\mathtt{k}} (x)+ x \frac{d}{dx}\mathtt{W}_{\nu , c}^{\mathtt{k}} (x) \\ &\quad =\sum_{r=0}^{\infty }\frac{(-c)^{r} ( 2r+\frac{\nu }{\mathtt{k}} ) ^{2} }{ \Gamma_{\mathtt{k}}(r \mathtt{k}+ \nu + \mathtt{k}) r!} \biggl( \frac{x}{2} \biggr) ^{2r+\frac{\nu }{\mathtt{k}}} \\ &\quad = \sum_{r=1}^{\infty }\frac{(-c)^{r} 4r ( r+\frac{\nu }{ \mathtt{k}} ) }{ \Gamma_{\mathtt{k}}(r \mathtt{k}+ \nu + \mathtt{k}) r!} \biggl( \frac{x}{2} \biggr) ^{2r+\frac{\nu }{\mathtt{k}}} \\ &\quad \quad {}+ \frac{\nu^{2}}{\mathtt{k}^{2}} \sum _{r=0}^{\infty }\frac{(-c)^{r} }{ \Gamma_{\mathtt{k}}(r \mathtt{k}+ \nu + \mathtt{k}) r!} \biggl( \frac{x}{2} \biggr) ^{2r+\frac{\nu }{\mathtt{k}}} \\ &\quad =\frac{4}{\mathtt{k}} \sum_{r=1}^{\infty } \frac{(-c)^{r} }{ \Gamma_{\mathtt{k}}(r \mathtt{k}+ \nu ) (r-1)!} \biggl( \frac{x}{2} \biggr) ^{2r+\frac{\nu }{\mathtt{k}}} + \frac{\nu^{2}}{\mathtt{k}^{2}} \mathtt{W}_{\nu , c}^{\mathtt{k}} (x) \\ &\quad =- \frac{c x^{2}}{\mathtt{k}}\mathtt{W}_{\nu , c}^{\mathtt{k}} (x) + \frac{\nu^{2}}{\mathtt{k}^{2}}\mathtt{W}_{\nu , c}^{\mathtt{k}} (x). \end{aligned}$$

A further simplification leads to the differential equation (2.1). □

### Recurrence relations

From (2.2) we have

$$\begin{aligned} x \frac{d}{dx}\mathtt{W}_{\nu , c}^{\mathtt{k}} (x) &= \frac{1}{ \mathtt{k}}\sum_{r=0}^{\infty } \frac{(-c)^{r} ( 2r \mathtt{k} + {\nu } ) }{ \Gamma_{\mathtt{k}}(r \mathtt{k}+ \nu + \mathtt{k}) r!} \biggl( \frac{x}{2} \biggr) ^{2r+\frac{\nu }{\mathtt{k}}} \\ & =\frac{\nu }{\mathtt{k}}\sum_{r=0}^{\infty }\frac{(-c)^{r} }{ \Gamma_{\mathtt{k}}(r \mathtt{k}+ \nu + \mathtt{k}) r!} \biggl( \frac{x}{2} \biggr) ^{2r+\frac{\nu }{\mathtt{k}}} \\ &\quad {}+2 \sum _{r=1}^{\infty }\frac{(-c)^{r}}{ \Gamma_{\mathtt{k}}(r \mathtt{k}+ \nu + \mathtt{k}) (r-1)!} \biggl( \frac{x}{2} \biggr) ^{2r+\frac{\nu }{\mathtt{k}}} \\ & =\frac{\nu }{\mathtt{k}}\mathtt{W}_{\nu , c}^{\mathtt{k}}(x) +2 \sum _{r=0}^{\infty }\frac{(-c)^{r+1}}{ \Gamma_{\mathtt{k}}(r \mathtt{k}+ \nu + 2\mathtt{k}) r!} \biggl( \frac{x}{2} \biggr) ^{2r+2+\frac{ \nu }{\mathtt{k}}} \\ & =\frac{\nu }{\mathtt{k}}\mathtt{W}_{\nu , c}^{\mathtt{k}}(x)- xc \mathtt{W}_{\nu +\mathtt{k}, c}^{\mathtt{k}}(x). \end{aligned}$$

Thus we have the difference equation

2.3 $$\begin{aligned} x \frac{d}{dx}\mathtt{W}_{\nu , c}^{\mathtt{k}} (x)= \frac{\nu }{ \mathtt{k}}\mathtt{W}_{\nu , c}^{\mathtt{k}}(x)- xc \mathtt{W}_{ \nu +\mathtt{k}, c}^{\mathtt{k}}(x). \end{aligned}$$

Again, rewrite (2.2) as

$$\begin{aligned} x \frac{d}{dx}\mathtt{W}_{\nu , c}^{\mathtt{k}} (x) &= \frac{1}{ \mathtt{k}}\sum_{r=0}^{\infty } \frac{(-c)^{r} ( 2r \mathtt{k} +2 {\nu } ) - \nu }{ \Gamma_{\mathtt{k}}(r \mathtt{k}+ \nu + \mathtt{k}) r!} \biggl( \frac{x}{2} \biggr) ^{2r+\frac{\nu }{\mathtt{k}}} \\ &=- \frac{\nu }{\mathtt{k}}\sum_{r=0}^{\infty }\frac{(-c)^{r}}{\Gamma_{\mathtt{k}}(r \mathtt{k}+ \nu + \mathtt{k}) r!} \biggl( \frac{x}{2} \biggr) ^{2r+\frac{\nu }{\mathtt{k}}} +2\sum _{r=0}^{\infty }\frac{(-c)^{r} ( r \mathtt{k} +{\nu } ) }{ \Gamma_{\mathtt{k}}(r \mathtt{k}+ \nu + \mathtt{k}) r!} \biggl( \frac{x}{2} \biggr) ^{2r+\frac{\nu }{ \mathtt{k}}} \\ &=- \frac{\nu }{\mathtt{k}} \mathtt{W}_{\nu , c}^{\mathtt{k}} (x) + \frac{x}{ \mathtt{k}} \sum_{r=0}^{\infty } \frac{(-c)^{r}}{ \Gamma_{\mathtt{k}}(r \mathtt{k}+ \nu - \mathtt{k} + \mathtt{k}) r!} \biggl( \frac{x}{2} \biggr) ^{2r+\frac{\nu -\mathtt{k}}{\mathtt{k}}} \\ &=- \frac{\nu }{\mathtt{k}} \mathtt{W}_{\nu , c}^{\mathtt{k}} (x) + \frac{x}{ \mathtt{k}} \mathtt{W}_{\nu -\mathtt{k}, c}^{\mathtt{k}} (x). \end{aligned}$$

This gives us the second difference equation

2.4 $$\begin{aligned} x \frac{d}{dx}\mathtt{W}_{\nu , c}^{\mathtt{k}} (x) = \frac{x}{ \mathtt{k}} \mathtt{W}_{\nu -\mathtt{k}, c}^{\mathtt{k}} (x) - \frac{ \nu }{\mathtt{k}} \mathtt{W}_{\nu , c}^{\mathtt{k}} (x). \end{aligned}$$

Thus (2.3) and (2.4) lead to the following recurrence relations.

#### Proposition 2.2

*Let* $\mathtt{k}>0$ *and* $\nu > -\mathtt{k}$. *Then*

2.5 $$\begin{aligned}& 2 \nu \mathtt{W}_{\nu , c}^{\mathtt{k}}(x) =x \mathtt{W}_{\nu - \mathtt{k}, c}^{\mathtt{k}} (x) + xc \mathtt{k} \mathtt{W}_{\nu + \mathtt{k}, c}^{\mathtt{k}}(x), \end{aligned}$$

2.6 $$\begin{aligned}& \mathtt{W}^{\mathtt{k}}_{\nu -\mathtt{k}, c}(x) =\frac{2}{x} \sum_{r=0} ^{\infty }(-1)^{r} (\nu + 2 r \mathtt{k}) \mathtt{W}^{\mathtt{k}}_{ \nu + 2r \mathtt{k}, c}(x), \end{aligned}$$

2.7 $$\begin{aligned}& \frac{d}{dx} \bigl( x^{\frac{\nu }{\mathtt{k}}} \mathtt{W}^{ \mathtt{k}}_{\nu , c}(x) \bigr) =\frac{x^{\frac{\nu }{\mathtt{k}}}}{ \mathtt{k}} \mathtt{W}^{\mathtt{k}}_{\nu -\mathtt{k}, c}(x), \end{aligned}$$

2.8 $$\begin{aligned}& \frac{d}{dx} \bigl( x^{-\frac{\nu }{\mathtt{k}}} \mathtt{W}^{ \mathtt{k}}_{\nu , c}(x) \bigr) =-c x^{-\frac{\nu }{\mathtt{k}}} \mathtt{W}^{\mathtt{k}}_{\nu +\mathtt{k}, c}(x), \end{aligned}$$

2.9 $$\begin{aligned}& \frac{d^{m}}{dx^{m}} \bigl( \mathtt{W}^{\mathtt{k}}_{\nu , c}(x) \bigr) = \frac{1}{2^{m} \mathtt{k}^{m}} \sum_{n=0}^{m} (-1)^{n} \left ( \textstyle\begin{array}{@{}c@{}} m \\ n \end{array}\displaystyle \right ) c^{n} \mathtt{k}^{n} \mathtt{W}^{\mathtt{k}}_{\nu -m \mathtt{k}+2 n \mathtt{k}, c}(x) \quad \textit{for all } m \in \mathbb{N}. \end{aligned}$$

#### Proof

Relation (2.5) follows by subtracting (2.4) from (2.3).

Next to establish (2.6), let us rewrite (2.5) as

2.10 $$\begin{aligned} \mathtt{W}_{\nu -\mathtt{k}, c}^{\mathtt{k}} (x) + c \mathtt{k} \mathtt{W}_{\nu +\mathtt{k}, c}^{\mathtt{k}}(x) = 2 \frac{\nu }{x} \mathtt{W}_{\nu , c}^{\mathtt{k}}(x). \end{aligned}$$

Now multiply both sides of (2.10) by $-c \mathtt{k}$ and replace *ν* by $\nu +2\mathtt{k}$. Then we have

2.11 $$\begin{aligned} - c \mathtt{k} \mathtt{W}_{\nu +\mathtt{k}, c}^{\mathtt{k}} (x) -c ^{2} \mathtt{k}^{2} \mathtt{W}_{\nu +3 \mathtt{k}, c}^{\mathtt{k}}(x) = -2 c \mathtt{k} \frac{\nu +2 \mathtt{k}}{x}\mathtt{W}_{\nu +2 \mathtt{k}, c}^{\mathtt{k}}(x). \end{aligned}$$

Similarly, multiplying both sides of (2.10) by $c^{2} \mathtt{k}^{2}$ and replacing *ν* by $\nu +4\mathtt{k}$ give

2.12 $$\begin{aligned} c^{2} \mathtt{k}^{2} \mathtt{W}_{\nu +3 \mathtt{k}, c}^{\mathtt{k}} (x) +c^{3} \mathtt{k}^{3} \mathtt{W}_{\nu +5 \mathtt{k}, c}^{\mathtt{k}}(x) = 2 c^{2}\mathtt{k}^{2} \frac{\nu +4 \mathtt{k}}{x} \mathtt{W}_{\nu +4 \mathtt{k}, c}^{\mathtt{k}}(x). \end{aligned}$$

Continuing and adding them lead to (2.6).

From definition (1.9) it is clear that

2.13 $$\begin{aligned} x^{\frac{\nu }{\mathtt{k}}}\mathtt{W}_{\nu , c}^{\mathtt{k}} (x) = \sum_{r=0}^{\infty }\frac{(-c)^{r} }{ \Gamma_{\mathtt{k}}(r \mathtt{k}+ \nu + \mathtt{k})2^{2r+\frac{\nu }{\mathtt{k}}} r!} ( x ) ^{2r+\frac{2\nu }{\mathtt{k}}}. \end{aligned}$$

The derivative of (2.13) with respect to *x* is

$$\begin{aligned} \frac{d}{dx} \bigl( x^{\frac{\nu }{\mathtt{k}}}\mathtt{W}_{\nu , c}^{ \mathtt{k}} (x) \bigr) &=\sum_{r=0}^{\infty } \frac{(-c)^{r} (2r+\frac{2 \nu }{\mathtt{k}}) }{ \Gamma_{\mathtt{k}}(r \mathtt{k}+ \nu + \mathtt{k})2^{2r+\frac{\nu }{\mathtt{k}}} r!} ( x ) ^{2r+\frac{2 \nu }{\mathtt{k}}-1} \\ &=\frac{x^{\frac{\nu }{\mathtt{k}}}}{\mathtt{k}}\sum_{r=0}^{\infty } \frac{(-c)^{r} }{ \Gamma_{\mathtt{k}}(r \mathtt{k}+ \nu ) r!} \biggl( \frac{x}{2} \biggr) ^{2r+\frac{\nu }{\mathtt{k}}-1} \\ &= \frac{x^{\frac{\nu }{\mathtt{k}}}}{ \mathtt{k}}\mathtt{W}_{\nu -\mathtt{k}, c}^{\mathtt{k}} (x). \end{aligned}$$

Similarly,

$$\begin{aligned} \frac{d}{dx} \bigl( x^{-\frac{\nu }{\mathtt{k}}}\mathtt{W}_{\nu , c} ^{\mathtt{k}} (x) \bigr) &=\sum_{r=1}^{\infty } \frac{(-c)^{r} 2r }{ \Gamma_{\mathtt{k}}(r \mathtt{k}+ \nu + \mathtt{k})2^{2r+\frac{\nu }{ \mathtt{k}}} r!} ( x ) ^{2r-1} \\ &=x^{-\frac{\nu }{\mathtt{k}}}\sum_{r=1}^{\infty } \frac{(-c)^{r} }{ \Gamma_{\mathtt{k}}(r \mathtt{k}+ \nu +\mathtt{k}) (r-1)!} \biggl( \frac{x}{2} \biggr) ^{2r+\frac{\nu }{\mathtt{k}}-1} \\ &=x^{-\frac{\nu }{\mathtt{k}}}\sum_{r=0}^{\infty } \frac{(-c)^{r+1} }{ \Gamma_{\mathtt{k}}(r \mathtt{k}+ \nu +2\mathtt{k}) r!} \biggl( \frac{x}{2} \biggr) ^{2r+\frac{\nu }{\mathtt{k}}+1} \\ & =-c x^{-\frac{\nu }{\mathtt{k}}} \mathtt{W}_{\nu +\mathtt{k}, c}^{\mathtt{k}} (x). \end{aligned}$$

Identity (2.9) can be proved by using mathematical induction on *m*. Recall that

$$ \left ( \textstyle\begin{array}{@{}c@{}} r \\ r \end{array}\displaystyle \right ) =\left ( \textstyle\begin{array}{@{}c@{}} r \\ 0 \end{array}\displaystyle \right ) =1 $$

and

$$ \left ( \textstyle\begin{array}{@{}c@{}} r \\ n \end{array}\displaystyle \right ) +\left ( \textstyle\begin{array}{@{}c@{}} r \\ n-1 \end{array}\displaystyle \right ) = \left ( \textstyle\begin{array}{@{}c@{}} r+1 \\ n \end{array}\displaystyle \right ) . $$

For $m=1$, the proof of identity (2.9) is equivalent to showing that

2.14 $$\begin{aligned} 2 \mathtt{k} \frac{d}{dx}\mathtt{W}_{\nu , c}^{\mathtt{k}} (x) &= \mathtt{W}_{\nu -\mathtt{k}, c}^{\mathtt{k}} (x) - c \mathtt{k} \mathtt{W}_{\nu +\mathtt{k}, c}^{\mathtt{k}}(x). \end{aligned}$$

This relation can be obtained by simply adding (2.3) and (2.4). Thus, identity (2.9) holds for $m=1$.

Assume that identity (2.9) also holds for any $m=r \geq 2$, that is,

$$\begin{aligned} \frac{d^{r}}{dx^{r}} \bigl( \mathtt{W}^{\mathtt{k}}_{\nu , c}(x) \bigr) &= \frac{1}{2^{m} \mathtt{k}^{r}} \sum_{n=0}^{r} (-1)^{n} \left ( \textstyle\begin{array}{@{}c@{}} r \\ n \end{array}\displaystyle \right ) c^{n} \mathtt{k}^{n} \mathtt{W}^{\mathtt{k}}_{\nu -r \mathtt{k}+2 n \mathtt{k}, c}(x). \end{aligned}$$

This implies, for $m=r+1$,

$$\begin{aligned} &\frac{d^{r+1}}{dx^{r+1}} \bigl( \mathtt{W}^{\mathtt{k}}_{\nu , c}(x) \bigr) \\ &\quad = \frac{1}{2^{r} \mathtt{k}^{r}} \sum_{n=0}^{r} (-1)^{n} \left ( \textstyle\begin{array}{@{}c@{}} r \\ n \end{array}\displaystyle \right ) c^{n} \mathtt{k}^{n} \frac{d}{dr}\mathtt{W}^{\mathtt{k}}_{ \nu -r \mathtt{k}+2 n \mathtt{k}, c}(x) \\ &\quad = \frac{1}{2^{r+1} \mathtt{k}^{r+1}} \sum_{n=0}^{r} (-1)^{n} \left ( \textstyle\begin{array}{@{}c@{}} r \\ n \end{array}\displaystyle \right ) c^{n} \mathtt{k}^{n} \bigl(\mathtt{W}^{\mathtt{k}}_{\nu -(r+1) \mathtt{k}+2 n \mathtt{k}, c}(x)-c \mathtt{k}\mathtt{W}^{\mathtt{k}} _{\nu -(r-1) \mathtt{k}+2 n \mathtt{k}, c}(x) \bigr) \\ &\quad = \frac{1}{2^{r+1} \mathtt{k}^{r+1}} \sum_{n=0}^{r} (-1)^{n} \left ( \textstyle\begin{array}{@{}c@{}} r \\ n \end{array}\displaystyle \right ) c^{n} \mathtt{k}^{n} \mathtt{W}^{\mathtt{k}}_{\nu -(r+1) \mathtt{k}+2 n \mathtt{k}, c}(x) \\ & \quad\quad {}- \frac{1}{2^{r+1} \mathtt{k}^{r+1}} \sum_{n=0}^{r} (-1)^{n} \left ( \textstyle\begin{array}{@{}c@{}} r \\ n \end{array}\displaystyle \right ) c^{n+1} \mathtt{k}^{n+1}\mathtt{W}^{\mathtt{k}}_{\nu -(r-1) \mathtt{k}+2 n \mathtt{k}, c}(x) \\ &\quad =\frac{1}{2^{r+1} \mathtt{k}^{r+1}} \Biggl[ \mathtt{W}^{\mathtt{k}} _{\nu -(r+1) \mathtt{k}, c}(x) +\sum _{n=1}^{r} (-1)^{r} \left ( \left ( \textstyle\begin{array}{@{}c@{}} r \\ n \end{array}\displaystyle \right ) +\left ( \textstyle\begin{array}{@{}c@{}} r \\ n-1 \end{array}\displaystyle \right ) \right ) \mathtt{W}^{\mathtt{k}}_{\nu -(r+1) \mathtt{k}+2n \mathtt{k}, c}(x) \\ & \quad \quad {} -(-1)^{r} c^{r+1}\mathtt{k}^{r+1} \mathtt{W}^{\mathtt{k}}_{ \nu +(r+1) \mathtt{k}, c}(x) \Biggr] \\ &\quad =\frac{1}{2^{r+1} \mathtt{k}^{r+1}} \Biggl[ \left ( \textstyle\begin{array}{@{}c@{}} r+1 \\ 0 \end{array}\displaystyle \right ) \mathtt{W}^{\mathtt{k}}_{\nu -(r+1) \mathtt{k}, c}(x) \\ &\quad \quad {}+\sum_{n=1}^{r} (-1)^{r} \left (\textstyle\begin{array}{@{}c@{}} r+1 \\ n \end{array}\displaystyle \right ) \mathtt{W}^{\mathtt{k}}_{\nu -(r+1) \mathtt{k}+2n \mathtt{k}, c}(x) \\ & \quad \quad {} +(-1)^{r+1} \left ( \textstyle\begin{array}{@{}c@{}} r+1 \\ r+1 \end{array}\displaystyle \right ) c^{r+1} \mathtt{k}^{r+1}\mathtt{W}^{\mathtt{k}}_{\nu -(r+1) \mathtt{k}+2(r+1) \mathtt{k}, c}(x) \Biggr] \\ &\quad =\frac{1}{2^{r+1} \mathtt{k}^{r+1}} \sum_{n=0}^{r+1} (-1)^{r} \left ( \textstyle\begin{array}{@{}c@{}} r+1 \\ n \end{array}\displaystyle \right ) \mathtt{W}^{\mathtt{k}}_{\nu -(r+1) \mathtt{k}+2n \mathtt{k},c}(x). \end{aligned}$$

Hence, identity (2.9) is concluded by the mathematical induction on *m*. □

### Integral representations of k-Bessel functions

Now we will derive two integral representations of the functions $\mathtt{W}_{\nu , c}^{\mathtt{k}}$. For this purpose, we need to recall the k-Beta functions from [15]. The k version of the beta functions is defined by

2.15 $$\begin{aligned} \mathtt{B}_{\mathtt{k}}(x, y)=\frac{ \Gamma_{\mathtt{k}}(x) \Gamma_{\mathtt{k}}(y)}{\Gamma_{\mathtt{k}}(x+y)}= \frac{1}{\mathtt{k}} \int_{0}^{1} t^{\frac{x}{\mathtt{k}}-1}(1-t)^{\frac{y}{ \mathtt{k}}-1}\,dt. \end{aligned}$$

Substituting *t* by $t^{2}$ on the integral in (2.15), it follows that

2.16 $$\begin{aligned} \mathtt{B}_{\mathtt{k}}(x, y)=\frac{2}{\mathtt{k}} \int_{0}^{1} t^{\frac{2x}{ \mathtt{k}}-1} \bigl(1-t^{2} \bigr)^{\frac{y}{\mathtt{k}}-1}\,dt. \end{aligned}$$

Let $x=(r+1) \mathtt{k}$ and $y=\nu $. Then from (2.15) and (2.16) we have

2.17 $$\begin{aligned} \frac{ 1}{\Gamma_{\mathtt{k}}(r \mathtt{k}+\nu +\mathtt{k})}=\frac{2}{ \Gamma_{\mathtt{k}}((r+1) \mathtt{k}) \Gamma_{\mathtt{k}}(\nu )} \int _{0}^{1} t^{2r+1} \bigl(1-t^{2} \bigr)^{\frac{\nu }{\mathtt{k}}-1}\,dt. \end{aligned}$$

According to [15], we have the identity $\Gamma_{\mathtt{k}}( \mathtt{k} x)= \mathtt{k}^{x-1} \Gamma (x)$. This gives

2.18 $$\begin{aligned} \frac{ 1}{\Gamma_{\mathtt{k}}(r \mathtt{k}+\nu +\mathtt{k})}=\frac{2}{ \mathtt{k}^{r}\Gamma (r+1) \Gamma_{\mathtt{k}}(\nu )} \int_{0}^{1} t ^{2r+1} \bigl(1-t^{2} \bigr)^{\frac{\nu }{\mathtt{k}}-1}\,dt. \end{aligned}$$

Now (1.9) and (2.18) together yield the first integral representation

2.19 $$\begin{aligned} \mathtt{W}_{\nu , c}^{\mathtt{k}} (x) & = \frac{2}{\Gamma_{\mathtt{k}}( \nu )} \biggl( \frac{x }{2} \biggr) ^{\frac{\nu }{\mathtt{k}}} \int_{0} ^{1} t \bigl(1-t^{2} \bigr)^{\frac{\nu }{\mathtt{k}}-1} \sum_{r=0}^{\infty } \frac{(-c)^{r} }{ \Gamma (r+1) r!} \biggl( \frac{x t}{2 \sqrt{\mathtt{k}}} \biggr) ^{2r}\,dt \\ &=\frac{2}{\Gamma_{\mathtt{k}}(\nu )} \biggl( \frac{x }{2} \biggr) ^{\frac{ \nu }{\mathtt{k}}} \int_{0}^{1} t \bigl(1-t^{2} \bigr)^{\frac{\nu }{\mathtt{k}}-1} \mathcal{W}_{0, 1, c} \biggl( \frac{x t}{\sqrt{\mathtt{k}}} \biggr)\,dt, \end{aligned}$$

where $\mathcal{W}_{p, b, c}$ is defined in (1.8).

For the second integral representation, substitute $x= r+\mathtt{k}/2$ and $y= \nu +\mathtt{k}/2$ into (2.16). Then (2.17) can be rewritten as

2.20 $$\begin{aligned} \frac{ 1}{\Gamma_{\mathtt{k}}(r \mathtt{k}+\nu +\mathtt{k})}=\frac{2}{ \Gamma_{\mathtt{k}} ( ( r+\frac{1}{2} ) \mathtt{k} ) \Gamma_{\mathtt{k}} ( \nu +\frac{\mathtt{k}}{2} ) } \int_{0} ^{1} t^{2r} \bigl(1-t^{2} \bigr)^{\frac{\nu }{\mathtt{k}}-\frac{1}{2}}\,dt. \end{aligned}$$

Again, the identity $\Gamma_{\mathtt{k}}(\mathtt{k} x)= \mathtt{k} ^{x-1} \Gamma (x)$ yields

2.21 $$\begin{aligned} \Gamma_{\mathtt{k}} \biggl( \biggl( r+\frac{1}{2} \biggr) \mathtt{k} \biggr) =\mathtt{k}^{r-\frac{1}{2}}\Gamma \biggl( r+\frac{1}{2} \biggr) . \end{aligned}$$

Further, the Legendre duplication formula (see [4, 6])

2.22 $$ \Gamma {(z)}\Gamma { \biggl( z+ \frac{1}{2} \biggr) }= 2^{1-2z} \sqrt{\pi } \Gamma {(2z)} $$

shows that

$$ \Gamma \biggl( r+\frac{1}{2} \biggr) r!= r \Gamma \biggl( r+ \frac{1}{2} \biggr) \Gamma (r)= \frac{\sqrt{\pi } (2r)!}{2^{2r}}. $$

This, together with (2.20) and (2.21), reduces the series (1.9) of $\mathtt{W}^{\mathtt{k}}_{\nu , c}$ to

2.23 $$\begin{aligned} \mathtt{W}_{\nu , c}^{\mathtt{k}} (x) & = \frac{2\sqrt{\mathtt{k}}}{ \Gamma_{\mathtt{k}} ( \nu +\frac{\mathtt{k}}{2} ) } \biggl( \frac{x }{2} \biggr) ^{\frac{\nu }{\mathtt{k}}} \int_{0}^{1} \bigl(1-t^{2} \bigr)^{\frac{ \nu }{\mathtt{k}}-\frac{1}{2}} \sum_{r=0}^{\infty } \frac{(-c)^{r} }{ \Gamma (r+1) r!} \biggl( \frac{x t}{2 \sqrt{\mathtt{k}}} \biggr) ^{2r}\,dt \\ &=\frac{2\sqrt{\mathtt{k}}}{\sqrt{\pi }\Gamma_{\mathtt{k}} ( \nu +\frac{ \mathtt{k}}{2} ) } \biggl( \frac{x }{2} \biggr) ^{\frac{\nu }{ \mathtt{k}}} \int_{0}^{1} \bigl(1-t^{2} \bigr)^{\frac{\nu }{\mathtt{k}}- \frac{1}{2}} \sum_{r=0}^{\infty } \frac{(-c)^{r} }{ (2r)!} \biggl( \frac{x t}{ \sqrt{\mathtt{k}}} \biggr) ^{2r}\,dt. \end{aligned}$$

Finally, for $c=\pm \alpha^{2}$, $\alpha \in \mathbb{R}$, representation (2.23) respectively leads to

2.24 $$\begin{aligned} \mathtt{W}_{\nu , \alpha^{2}}^{\mathtt{k}} (x) =\frac{2\sqrt{ \mathtt{k}}}{\sqrt{\pi }\Gamma_{\mathtt{k}} ( \nu +\frac{ \mathtt{k}}{2} ) } \biggl( \frac{x }{2} \biggr) ^{\frac{\nu }{ \mathtt{k}}} \int_{0}^{1} \bigl(1-t^{2} \bigr)^{\frac{\nu }{\mathtt{k}}- \frac{1}{2}} \cos \biggl( \frac{\alpha x t}{ \sqrt{\mathtt{k}}} \biggr)\,dt \end{aligned}$$

and

2.25 $$\begin{aligned} \mathtt{W}_{\nu , -\alpha^{2}}^{\mathtt{k}} (x) =\frac{2\sqrt{ \mathtt{k}}}{\sqrt{\pi }\Gamma_{\mathtt{k}} ( \nu +\frac{ \mathtt{k}}{2} ) } \biggl( \frac{x }{2} \biggr) ^{\frac{\nu }{ \mathtt{k}}} \int_{0}^{1} \bigl(1-t^{2} \bigr)^{\frac{\nu }{\mathtt{k}}- \frac{1}{2}} \cosh \biggl( \frac{\alpha x t}{ \sqrt{\mathtt{k}}} \biggr)\,dt. \end{aligned}$$

#### Example 2.1

If $\nu =\mathtt{k}/2$, then from (2.24) computations give the relation between sine and generalized k-Bessel functions by

$$ \sin \biggl( \frac{\alpha x}{\sqrt{\mathtt{k}}} \biggr) = \frac{\alpha }{\mathtt{k}}\sqrt{ \frac{\pi x}{2}}\mathtt{W}_{\frac{\nu }{ \mathtt{k}}, \alpha^{2}}^{\mathtt{k}} (x). $$

Similarly, the relation

$$ \sinh \biggl( \frac{\alpha x}{\sqrt{\mathtt{k}}} \biggr) = \frac{\alpha }{\mathtt{k}}\sqrt{ \frac{\pi x}{2}}\mathtt{W}_{\frac{\nu }{ \mathtt{k}}, -\alpha^{2}}^{\mathtt{k}} (x) $$

can be derived from (2.25).

## Monotonicity and log-convexity properties

This section is devoted to discuss the monotonicity and log-convexity properties of the modified k-Bessel function $\mathtt{W} _{\nu , -1}^{\mathtt{k}}=\mathtt{I}_{\nu }^{\mathtt{k}}$. As consequences of those results, we derive several functional inequalities for $\mathtt{I}_{\nu }^{\mathtt{k}}$.

The following result of Biernacki and Krzyż [7] will be required.

### Lemma 3.1

([7])

*Consider the power series* $f(x)=\sum_{k=0} ^{\infty }a_{k} x^{k}$ *and* $g(x)=\sum_{k=0}^{\infty }b_{k} x^{k}$, *where* $a_{k} \in \mathbb{R}$ *and* $b_{k} > 0$ *for all k*. *Further*, *suppose that both series converge on* $\vert x \vert < r$. *If the sequence* $\{a_{k}/b_{k} \}_{k\geq 0}$ *is increasing* (*or decreasing*), *then the function* $x \mapsto f(x)/g(x)$ *is also increasing* (*or decreasing*) *on* $(0,r)$.

The lemma still holds when both *f* and *g* are even or both are odd functions.

We now state and prove our main results in this section. Consider the functions

3.1 $$\begin{aligned} \mathcal{I}_{\nu }^{\mathtt{k}}(x):= \biggl( \frac{2}{x} \biggr) ^{\frac{ \nu }{\mathtt{k}}}\Gamma_{\mathtt{k}} (\nu +\mathtt{k}) \mathtt{I} _{\nu }^{\mathtt{k}}(x)=\sum_{r=0}^{\infty }f_{r}( \nu ) x^{2r}, \end{aligned}$$

where

3.2 $$\begin{aligned} \begin{aligned} & \mathtt{I}_{\nu }^{\mathtt{k}}(x)= \mathtt{W}_{\nu , -1}^{\mathtt{k}} (x) =\sum_{r=0}^{\infty }\frac{1 }{ \Gamma_{\mathtt{k}}(r \mathtt{k}+ \nu + \mathtt{k}) r!} \biggl( \frac{x}{2} \biggr) ^{2r+\frac{\nu }{ \mathtt{k}}} \quad \mbox{and} \\ &f_{r}(\nu )= \frac{\Gamma_{ \mathtt{k}}{(\nu +\mathtt{k})}}{\Gamma_{\mathtt{k}}{(r{\mathtt{k}}+ \nu +\mathtt{k})}4^{r} r!}. \end{aligned} \end{aligned}$$

Then we have the following properties.

### Theorem 3.1

*Let* $\mathtt{k}>0$. *The following results are true for the modified* k-*Bessel functions*:

- *If* $\nu \geq \mu >-\mathtt{k}$, *then the function* $x \mapsto {\mathcal{I}_{\mu }^{\mathtt{k}}(x)}/ {\mathcal{I}_{\nu } ^{\mathtt{k}}(x)}$ *is increasing on* $\mathbb{R}$.
- *The function* $\nu \mapsto \mathcal{I}^{\mathtt{k}}_{\nu + \mathtt{k}}(x) /\mathcal{I}^{\mathtt{k}}_{\nu }(x)$ *is increasing on* $(-\mathtt{k}, \infty )$, *that is*, *for* $\nu \geq \mu >-\mathtt{k}$,
  3.3 $$ \mathcal{I}_{\nu +\mathtt{k}}^{\mathtt{k}}(x)\mathcal{I}_{\mu }^{ \mathtt{k}}(x) \geq \mathcal{I}_{\nu }^{\mathtt{k}}(x)\mathcal{I}_{ \mu +\mathtt{k}}^{\mathtt{k}}(x) $$
  *for any fixed* $x>0$ *and* $\mathtt{k}>0$.
- *The function* $\nu \mapsto \mathcal{I}_{\nu }^{\mathtt{k}}(x)$ *is decreasing and log*-*convex on* $(-\mathtt{k}, \infty )$ *for each fixed* $x >0$.

### Proof

(a) From (3.1) it follows that

$$\begin{aligned} \frac{\mathcal{I}^{\mathtt{k}}_{\nu }(x)}{\mathcal{I}^{\mathtt{k}} _{\mu }(x)} =\frac{\sum_{r=0}^{\infty }f_{r}(\nu ) x^{2r}}{\sum_{r=0} ^{\infty }f_{r}(\mu ) x^{2r}}. \end{aligned}$$

Denote $w_{r}:=f_{r}(\nu )/f_{r}(\mu )$. Then

$$ w_{r}= \frac{\Gamma_{\mathtt{k}}{(\nu +\mathtt{k})}\Gamma_{\mathtt{k}} {(r{\mathtt{k}}+\mu +\mathtt{k})}}{\Gamma_{\mathtt{k}}{(\mu + \mathtt{k})} \Gamma_{\mathtt{k}}{(r{\mathtt{k}}+\nu +\mathtt{k})}}. $$

Now, using the property $\Gamma_{\mathtt{k}}{(y+\mathtt{k})}=y \Gamma_{\mathtt{k}}{(y)}$, we can show that

$$\begin{aligned} \frac{w_{r+1}}{w_{r}} &= \frac{\Gamma_{\mathtt{k}}{(r{\mathtt{k}}+ \nu +\mathtt{k})}\Gamma_{\mathtt{k}} {(r{\mathtt{k}}+\mu +2\mathtt{k})}}{ \Gamma_{\mathtt{k}}{(r{\mathtt{k}}+\mu +\mathtt{k})} \Gamma_{ \mathtt{k}}{(r{\mathtt{k}}+\nu +2\mathtt{k})}} =\frac{r{\mathtt{k}}+ \mu +\mathtt{k}}{r{\mathtt{k}}+\nu +\mathtt{k}}\leq 1 \end{aligned}$$

for all $\nu \geq \mu >-\mathtt{k}$. Hence, conclusion (a) follows from the Lemma 3.1.

(b) Let $\nu \geq \mu >-\mathtt{k}$. It follows from part (a) that

$$\begin{aligned} \frac{d}{dx} \biggl( \frac{\mathcal{I}^{\mathtt{k}}_{\nu }(x)}{ \mathcal{I}^{\mathtt{k}}_{\mu }(x)} \biggr) \geq 0 \end{aligned}$$

on $(0,\infty )$. Thus

3.4 $$\begin{aligned} \bigl( \mathcal{I}^{\mathtt{k}}_{\nu }(x) \bigr) ' \bigl( \mathcal{I} ^{\mathtt{k}}_{\mu }(x) \bigr) - \bigl( \mathcal{I}^{\mathtt{k}}_{ \nu }(x) \bigr) \bigl( \mathcal{I}^{\mathtt{k}}_{\mu }(x) \bigr) ' \geq 0. \end{aligned}$$

It now follows from (2.8) that

$$\begin{aligned} \frac{x}{2} \bigl(\mathcal{I}^{\mathtt{k}}_{\nu +k}(x) \mathcal{I} ^{\mathtt{k}}_{\mu }(x)-\mathcal{I}^{\mathtt{k}}_{\mu +k}(x) \mathcal{I}^{\mathtt{k}}_{\nu }(x) \bigr) \geq 0, \end{aligned}$$

whence $\mathcal{I}^{\mathtt{k}}_{\nu +k}/\mathcal{I}^{\mathtt{k}} _{\nu }$ is increasing for $\nu >-\mathtt{k}$ and for some fixed $x >0$, which concludes (b).

(c) It is clear that, for all $\nu >-\mathtt{k}$,

$$ f_{r}(\nu )= \frac{\Gamma_{\mathtt{k}}{(\nu +\mathtt{k})}}{ \Gamma_{\mathtt{k}}{(r{\mathtt{k}}+\nu +\mathtt{k})}4^{r} r!}>0. $$

A logarithmic differentiation of $f_{r}(\nu )$ with respect to *ν* yields

$$\begin{aligned} \frac{f_{r}'(\nu )}{f_{r}(\nu )}= \Psi_{\mathtt{k}}(\nu +\mathtt{k})- \Psi_{\mathtt{k}}(r \mathtt{k}+\nu +\mathtt{k})\leq 0 \end{aligned}$$

since $\Psi_{\mathtt{k}}$ are increasing functions on $(-\mathtt{k}, \infty )$. This implies that $f_{r}(\nu )$ is decreasing.

Thus, for $\mu \geq \nu >-\mathtt{k}$, it follows that

$$\begin{aligned} \sum_{r=0}^{\infty }f_{r}(\nu ) x^{2r} \geq \sum_{r=0}^{\infty }f_{r}( \mu ) x^{2r}, \end{aligned}$$

which is equivalent to say that the function $\nu \mapsto \mathcal{I} ^{\mathtt{k}}_{\nu }$ is decreasing on $(-\mathtt{k}, \infty )$ for some fixed $x >0$.

The twice logarithmic differentiation of $f_{r}(\nu )$ yields

$$\begin{aligned} \frac{\partial^{2}}{\partial \nu^{2}} (\log \bigl(f_{r}(\nu ) \bigr) &= \Psi_{k}'(\nu +\mathtt{k})-\Psi_{k}'(r \mathtt{k}+\nu +\mathtt{k}) \\ &=\sum_{n=0}^{\infty } \biggl( \frac{1}{(n\mathtt{k}+\nu +\mathtt{k})^{2}} - \frac{1}{(n\mathtt{k}+r\mathtt{k}+\nu +\mathtt{k})^{2}} \biggr) \\ &=\sum_{n=0}^{\infty }\frac{r \mathtt{k}(2n \mathtt{k}+ r \mathtt{k}+2 \nu +2\mathtt{k}) }{(n\mathtt{k}+\nu +\mathtt{k})^{2}(n\mathtt{k}+r \mathtt{k}+\nu +\mathtt{k})^{2}} \geq 0 \end{aligned}$$

for all $\mathtt{k}>0$ and $\nu > -\mathtt{k}$. Since, a sum of log-convex functions is log-convex, it follows that $\nu \to \mathcal{I}_{\nu }^{\mathtt{k}}$ is log-convex on $(-\mathtt{k}, \infty )$ for each fixed $x>0$. □

### Remark 3.1

One of the most significance consequences of the Theorem 3.1 is the Turán-type inequality for the function $\mathcal{I}_{\nu }^{\mathtt{k}}$. From the definition of log-convexity it follows from Theorem 3.1(c) that

$$\begin{aligned} \mathcal{I}_{\alpha \nu_{1}+(1-\alpha )\nu_{2}}^{\mathtt{k}}(x) \leq \bigl( \mathcal{I}_{\nu_{1}}^{\mathtt{k}} \bigr) ^{\alpha }(x) \bigl( \mathcal{I}_{\nu_{2}}^{\mathtt{k}} \bigr) ^{1-\alpha }(x), \end{aligned}$$

for $\alpha \in [0,1]$, $\nu_{1}, \nu_{2} > -\mathtt{k}$, and $x >0$. For any $a\in \mathbb{R}$ and $\nu \geq -k$, by choosing $\alpha =1/2, \nu_{1}=\nu -a$, and $\nu_{2}=\nu +a$, this inequality yields the reverse Turán-type inequality

3.5 $$\begin{aligned} \bigl( \mathcal{I}_{\nu }^{\mathtt{k}}(x) \bigr) ^{2} - \mathcal{I}_{ \nu -\mathtt{a}}^{\mathtt{k}}(x) \mathcal{I}_{\nu +\mathtt{a}}^{ \mathtt{k}}(x) \leq 0 \end{aligned}$$

for any $\nu \geq \vert a \vert -\mathtt{k}$.

Our final result is based on the Chebyshev integral inequality [26, p. 40], which states the following: suppose *f* and *g* are two integrable functions and monotonic in the same sense (either both decreasing or both increasing). Let $q: (a, b) \to \mathbb{R}$ be a positive integrable function. Then

3.6 $$\begin{aligned} \biggl( \int_{a}^{b} q(t) f(t)\,dt \biggr) \biggl( \int_{a}^{b} q(t) g(t)\,dt \biggr) \leq \biggl( \int_{a}^{b} q(t)\,dt \biggr) \biggl( \int_{a}^{b} q(t) f(t) g(t)\,dt \biggr) . \end{aligned}$$

Inequality (3.6) is reversed if *f* and *g* are monotonic in the opposite sense.

The following function is required:

3.7 $$\begin{aligned} \mathcal{J}_{\nu }^{\mathtt{k}}(x):= \biggl( \frac{2}{x} \biggr) ^{\frac{ \nu }{\mathtt{k}}}\Gamma_{\mathtt{k}} (\nu +\mathtt{k}) \mathtt{J} _{\nu }^{\mathtt{k}}(x)=\sum_{r=0}^{\infty }g_{r}( \nu ) x^{2r}, \end{aligned}$$

where

3.8 $$\begin{aligned} \begin{aligned} &\mathtt{J}_{\nu }^{\mathtt{k}}(x)= \mathtt{W}_{\nu , 1}^{\mathtt{k}} (x) =\sum_{r=0}^{\infty }\frac{(-1)^{r} }{ \Gamma_{\mathtt{k}}(r \mathtt{k}+ \nu + \mathtt{k}) r!} \biggl( \frac{x}{2} \biggr) ^{2r+\frac{ \nu }{\mathtt{k}}} \quad \text{and} \\ &g_{r}(\nu )= \frac{(-1)^{r} \Gamma_{\mathtt{k}}{(\nu +\mathtt{k})}}{\Gamma_{\mathtt{k}}{(r{\mathtt{k}}+ \nu +\mathtt{k})}4^{r} r!}. \end{aligned} \end{aligned}$$

### Theorem 3.2

*Let* $\mathtt{k}>0$. *Then*, *for* $\nu \in (-3\mathtt{k}/4,- \mathtt{k}/2] \cup [\mathtt{k}/2, \infty ) $,

3.9 $$\begin{aligned} \mathcal{I}_{\nu }^{\mathtt{k}}(x)\mathcal{I}_{\nu + \frac{\mathtt{k}}{2}}^{\mathtt{k}}(x) \leq \frac{\sqrt{\mathtt{k}}}{x} \sin \biggl( \frac{x}{\mathtt{k}} \biggr) \mathcal{I}_{2\nu +\frac{\mathtt{k}}{2}}^{\mathtt{k}}(x) \end{aligned}$$

*and*

3.10 $$\begin{aligned} \mathcal{J}_{\nu }^{\mathtt{k}}(x)\mathcal{J}_{\nu + \frac{\mathtt{k}}{2}}^{\mathtt{k}}(x) \leq \frac{\sqrt{\mathtt{k}}}{x} \sinh \biggl( \frac{x}{\mathtt{k}} \biggr) \mathcal{J}_{2\nu +\frac{\mathtt{k}}{2}}^{\mathtt{k}}(x). \end{aligned}$$

*Inequalities* (3.9) *and* (3.10) *are reversed if* $\nu \in (-\mathtt{k}/2, \mathtt{k}/2)$.

### Proof

Define the functions *q*, *f*, and *g* on $[0, 1]$ as

$$ q(t)= \cos \biggl( \frac{x t}{\sqrt{\mathtt{k}}} \biggr) , \qquad f(t)= \bigl(1-t ^{2} \bigr)^{\frac{v}{\mathtt{k}}-\frac{1}{2}}, \qquad g(t)= \bigl(1-t^{2} \bigr)^{\frac{v}{ \mathtt{k}}+\frac{1}{2}}. $$

Then, for any $x \geq 0$,

$$\begin{aligned}& \int_{0}^{1} q(t)\,dt = \int_{0}^{1} \cos \biggl( \frac{x t}{\sqrt{ \mathtt{k}}} \biggr)\,dt= \frac{\sqrt{\mathtt{k}}}{x} \sin \biggl( \frac{x }{\sqrt{\mathtt{k}}} \biggr) , \\& \int_{0}^{1} q(t) f(t)\,dt = \int_{0}^{1} \cos \biggl( \frac{x t}{\sqrt{ \mathtt{k}}} \biggr) \bigl(1-t^{2} \bigr)^{\frac{v}{\mathtt{k}}-\frac{1}{2}}\,dt= \mathcal{I}_{\nu }^{\mathtt{k}}(x) \quad \text{if } \nu \geq - \mathtt{k}, \\& \int_{0}^{1} q(t) g(t)\,dt = \int_{0}^{1} \cos \biggl( \frac{x t}{\sqrt{ \mathtt{k}}} \biggr) \bigl(1-t^{2} \bigr)^{\frac{v}{\mathtt{k}}+\frac{1}{2}}\,dt= \mathcal{I}_{\nu +\mathtt{k}}^{\mathtt{k}}(x) \quad \text{if } \nu \geq - 2\mathtt{k}, \\& \int_{0}^{1} q(t) f(t) g(t)\,dt = \int_{0}^{1} \cos \biggl( \frac{x t}{\sqrt{\mathtt{k}}} \biggr) \bigl(1-t^{2} \bigr)^{\frac{2 v}{\mathtt{k}}}\,dt= \mathcal{I}_{2 \nu +\frac{\mathtt{k}}{2}}^{\mathtt{k}}(x) \quad \text{if } \nu \geq - \frac{3\mathtt{k}}{4}. \end{aligned}$$

Since the functions *f* and *g* both are decreasing for $\nu \geq \mathtt{k}/2$ and both are increasing for $\nu \in (-3\mathtt{k}/4,- \mathtt{k}/2]$, inequality (3.6) yields (3.9). On the other hand, if $\nu \in (-\mathtt{k}/2, \mathtt{k}/2)$, then the function *f* is increasing, but *g* is decreasing, and hence inequality (3.9) is reversed.

Similarly, inequality (3.10) can be derived from (3.6) by choosing

$$ q(t)= \cosh \biggl( \frac{x t}{\sqrt{\mathtt{k}}} \biggr) , \qquad f(t)= \bigl(1-t ^{2} \bigr)^{\frac{v}{\mathtt{k}}-\frac{1}{2}}, \qquad g(t)= \bigl(1-t^{2} \bigr)^{\frac{v}{ \mathtt{k}}+\frac{1}{2}}. $$

 □

## Conclusion

It is shown that the generalized *k*-Bessel functions $W^{k}_{\nu ,c}$ are solutions of a second-order differential equation, which for $k=1$ is reduced to the well-known second-order Bessel differential equation. It is also proved that the generalized modified *k*-Bessel function $\mathcal{I}_{\nu }^{\mathtt{k}}$ is decreasing and log-convex on $(-\mathtt{k}, \infty )$ for each fixed $x >0$. Several other inequalities, especially the Turán-type inequality and reverse Turán-type inequality for $\mathcal{I}_{\nu }^{\mathtt{k}}$ are established.

Furthermore, we investigate the pattern for zeroes of $\mathcal{W} _{\nu }^{\mathtt{k}, 1}$ in two ways: (i) with respect to fixed k and variation of *ν* and (ii) with respect to fixed *ν* and variation of k.

From the data in Table 1 and Table 2, we can observe that the zeroes of $\mathtt{W}_{ \nu , 1}^{\mathtt{k}}$ are increasing in in both cases. However, we have no any analytical proof for this monotonicity of the zeroes of $W^{k}_{ \nu ,1}$. As there are several works on the zeroes of the classical Bessel functions, the zeroes of $\mathtt{W}_{ \nu , 1}^{\mathtt{k}}$ would be an interesting topic for future investigations. The monotonicity of the zeroes of $\mathtt{W}_{ \nu , c}^{\mathtt{k}}$ with respect to *c* and fixed k, *ν* will be another open problem for further studies.

**Table 1:** Positive zeroes of $\mathtt{W}_{ \nu , 1}^{\mathtt{k}}$ for fixed *ν* and different k

k	0.5	1	1.5	2	2.5
*ν* = −0.4 and *c* = 1					
1st zero	0.662422	1.75098	2.42334	2.95334	3.40423
2nd zero	2.96686	4.87852	6.24148	7.3588	8.32849
3rd zero	5.2018	8.01663	10.0812	11.7913	13.2836
*ν* = 0.5 and *c* = 1					
1st zero	2.70943	3.14159	3.55493	3.93277	4.28026
2nd zero	4.96077	6.28319	7.38858	8.35255	9.21757
3rd zero	7.19373	9.42478	11.2315	12.7879	14.1752

**Table 2:** Positive zeroes of $\mathtt{W}_{ \nu , 1}^{\mathtt{k}}$ for different *ν* and k

*ν*	−0.4	−0.3	0	0.5	1	1.5	2	2.5
k = 0.5 and *c* = 1								
1st zero	0.662422	0.97534	1.70047	2.70943	3.63143	4.51146	5.36577	6.20238
2nd zero	2.96686	3.21271	3.90328	4.96077	5.95189	6.90209	7.82393	8.72471
3rd zero	5.2018	5.43751	6.11911	7.19373	8.21647	9.20314	10.1629	11.1017
k = 1 and *c* = 1								
1st zero	1.75098	1.92285	2.40483	3.14159	3.83171	4.49341	5.13562	5.76346
2nd zero	4.87852	5.04213	5.52008	6.28319	7.01559	7.72525	8.41724	9.09501
3rd zero	8.01663	8.17785	8.65373	9.42478	10.1735	10.9041	11.6198	12.3229
k = 1.5 and *c* = 1								
1st zero	2.42334	2.55767	2.9453	3.55493	4.13426	4.69286	5.2362	5.76774
2nd zero	6.24148	6.37291	6.76069	7.38858	7.9979	8.5923	9.1744	9.74613
3rd zero	10.0812	10.2116	10.5986	11.2315	11.8513	12.4599	13.0587	13.6488
k = 2 and *c* = 1								
1st zero	2.95334	3.06754	3.40094	3.93277	4.44288	4.93703	5.41885	5.8908
2nd zero	7.3588	7.47176	7.80657	8.35255	8.88577	9.40825	9.92154	10.4269
3rd zero	11.7913	11.9037	12.2382	12.7879	13.3286	13.8616	14.3875	14.907
